# Chronic Non-Communicable Disease Burden among Recently Arrived Refugees in Syracuse, New York

**DOI:** 10.5334/aogh.5247

**Published:** 2026-10-15

**Authors:** Estee Wu, Madison Searles, Inijesu Taiwo, Christina D. Campagna, Moe Kuroda, Andrea V. Shaw

**Affiliations:** 1 Norton College of Medicine, State University of New York Upstate Medical University, Syracuse, NY 13210, United States; 2 Global Health Institute, State University of New York Upstate Medical University, 5010 Campuswood Drive, Suite 100 Campuswood, East Syracuse, NY 13057, United States; 3 Department of General Medicine, Toyama University Hospital, Toyama, Japan; 4 Department of Pediatrics, SUNY Upstate Medical University, NY 13210, United States; 5 Department of Internal Medicine, SUNY Upstate Medical University, NY 13210, United States

**Keywords:** health screening, refugee, non-communicable disease, hypertension, diabetes mellitus, heart rate, tachycardia

## Abstract

*Background:* Refugees resettling in the United States often come from regions with limited healthcare infrastructure. While communicable diseases are routinely screened for, non-communicable diseases (NCDs), including hypertension and diabetes mellitus, remain underrecognized. Lifetime adverse events contributing to chronic stress further increase cardiovascular disease risk in refugees.

*Objective:* To evaluate the chronic disease burden of blood pressure measurements in the hypertensive range and point-of-care capillary glucose values in the prediabetic and diabetic ranges among refugees arriving in Syracuse, New York and to examine how a community-based health screening program informs current U.S. refugee screening practices. Associations between elevated cardiometabolic measures (blood glucose, blood pressure, and heart rate) and demographic and clinical characteristics are explored.

*Methods:* We conducted a retrospective cross-sectional analysis of health screening data collected from recently arrived adult refugees in Syracuse, New York, between May 2023 and March 2025. Health screenings were conducted within the first few months of arrival and were offered to refugees aged 18 and older, with 529 total participants.

*Findings:* Participants were 55.8% female, with 69.8% of participants between 18 and 44 years old. Predominant regions of origin included the Eastern Mediterranean region (39.3%, including Somalia 14.2%, Syria 13.2%), the African region (36.1%, including Democratic Republic of Congo 17.8%), and region of the Americas (12.5%, including Venezuela 7.0%). Elevated heart rate was associated with glucose values in the diabetic range and gender. Glucose values in diabetic range were associated with blood pressure and age. Blood pressure was associated with age, gender, and BMI.

*Conclusions:* Our refugee cohort showed a notable burden of undiagnosed NCDs, including hypertension and hyperglycemia in the prediabetic/diabetic ranges. Statistically significant associations between cardiometabolic risk factors, including increased BMI, age, and gender, with chronic diseases underscore the need for continued interventions to improve access and uptake of preventive health services for refugees.

## 1. Introduction

The burden of chronic non-communicable diseases, particularly hypertension and diabetes mellitus, among refugees resettling in the United States (U.S.) is substantial and reflects the unique health challenges faced by this population. A cohort of refugees resettled to Buffalo, NY, demonstrated a prevalence of hypertension to be 24.1% and diabetes to be 7.8%, many with uncontrolled disease [1]. Among refugees enrolled in an intercultural psychiatry program, reported rates for both hypertension and diabetes were even higher –42% and 15.5%, respectively [2]. There are a multitude of risk factors that may contribute to the increased burden of hypertension and diabetes in refugee populations. These risk factors include obesity, age, gender, and country of origin [3]. Previous studies have indicated that obesity significantly increases the odds of developing diabetes (odds ratio [OR] = 2.49; 95% CI = 1.61–3.85) and hypertension (OR = 2.62; 95% CI = 1.92–3.58) among refugees [1]. Eastern European origin OR = 4.09; 95% CI = 2.00–8.38) and length of follow-up (OR = 1.06; 95% CI = 1.00–1.15), among other variables, were associated with hypertension [1]. In addition, increasing duration of residence in the U.S. has been linked to increased odds of developing both diabetes and hypertension, suggesting the influence of acculturation and lifestyle changes over time [3, 4]. People diagnosed with post-traumatic stress disorder (PTSD) and those who experience trauma are at increased risk for chronic diseases, including cardiovascular disease [5].

The American Diabetes Association highlights that refugee status itself is an independent risk factor for cardiovascular disease and notes that refugees are at a greater risk for obstacles against achieving optimal chronic disease management [6, 7]. These barriers include cultural and language barriers, limited access to transportation, food insecurity, and unfamiliarity with healthcare systems and community resources [7].

Current U.S. domestic refugee health guidance recommends a comprehensive Refugee Health Assessment (RHA) within 90 days following arrival to identify and manage medical conditions among newly resettled refugees [8]. The Community–Clinic Partnership (CCP) is a collaboration between an academic medical center and a refugee resettlement agency in Syracuse, NY, formalized in 2020 in response to increasing refugee community health needs. One of the joint outreach activities CCP engaged in was a community-based health screening program to assist with early detection of chronic disease (screening BMI, blood pressure, and blood sugar) and medical triage to guide RHA scheduling and facilitate linkage to healthcare. While the RHA focuses on routine review of overseas medical information and screening for infectious diseases, the health screening program is an opportunity for proactive outreach to identify at-risk patients who need expedited support.

In addition to screening measures including elevated blood pressure, blood glucose levels, and body mass index (BMI), heart rate was also evaluated. Tachycardia is associated with sympathetic overactivity, increased arterial wall stress, endothelial dysfunction, and the development of atherosclerotic plaques, making it an important cardiovascular risk factor [9]. Despite these well-established associations between tachycardia and adverse cardiovascular outcomes in literature, including hypertension, cardiovascular disease, sudden cardiac death, heart failure, stroke, and total cancer and mortality, the significance of elevated heart rate among refugee populations remains poorly characterized [10]. Tachycardia may function as a marker of heightened cardiovascular risk and may also reflect prolonged psychosocial stressors and comorbid disease [11–13]. It is hypothesized that tachycardia may be more common in refugee populations given the increased physiological arousal associated with PTSD, depression, and other psychosocial stressors. However, the prevalence and clinical significance of elevated heart rate including tachycardia, among refugee population, remains poorly characterized in existing literature [11, 14].

This paper aims to examine the prevalence of blood pressure measurements in the hypertensive range and point-of-care glucose values in the prediabetic and diabetic ranges among adult refugees participating in a community-based health screening program and descriptively compare these findings with estimates from the general U.S. population. The analysis draws on health screening data collected during a single screening encounter, including blood pressure, point-of-care glucose, heart rate, and basic demographic characteristics. Additionally, we aim to explore associations between risk factors, such as heart rate, age, gender, and BMI, and the presence of diabetes and hypertension. By characterizing elevated heart rate alongside other known cardiovascular risks, this paper seeks to better define the cardiovascular risk profile of refugees resettling in the U.S. Furthermore, we evaluate how this community-based health screening informs current U.S. refugee screening practices by early identification of cardiometabolic risk factors and triaged medical needs prior to a scheduled RHA.

## 2. Methods

### 2.1 Study design and settings

This project was conducted in Syracuse, NY. In 2024, New York welcomed over 6000 refugees, with the vast majority resettling in Upstate, New York, with over 30% resettling in Onondaga County, and with over 2000 resettled to Syracuse [15]. The most common countries of origin among these refugees include Afghanistan, Democratic Republic of Congo (DRC), and Syria [16, 17].

Since 2021, the State University of New York (SUNY) Upstate Medical University and Catholic Charities of Onondaga County (CCOC) Refugee Resettlement Services have established a CCP with the goal of improving health and wellness for local refugee populations through various projects [18]. Independent of any research objectives, Upstate Medical University faculty and students organized regular health screenings. Adults 18 years and older were offered BMI, fingerstick blood glucose, and blood pressure screening, with the goal of chronic disease screening, health education, and linkage to primary care resources. Health screenings conducted in the early days of resettlement, prior to a scheduled RHA, served as an opportunity to triage high-needs cases into earlier RHA appointments. This paper examined data collected through CCP, an initiative designed to improve health outcomes and facilitate outreach and disease surveillance of the refugee population in Syracuse, NY.

### 2.2 Data collection

A retrospective cross-sectional analysis was designed to assess the chronic disease burden of hypertension and diabetes mellitus on newly arrived refugees to Syracuse, NY, who participated in weekly health screenings at the CCOC refugee resettlement services community site. This initiative, Preventive Health Screenings for Refugees in the Community, was reviewed and granted exempt status by the SUNY Upstate Medical University Institutional Review Board (Project #2045075–2). A waiver of informed consent was approved because the project involved no more than minimal risk and comprised a secondary analysis of preexisting, de-identified data. Various translation and interpretation methods were used, including linguistically congruent case managers, health navigators, and phone interpretation services. The dataset did not include direct personal identifiers or unique codes that could be used to identify individual participants. A total of 529 health screenings were conducted between May 2023 and March 2025. Data were collected on paper and manually entered into the REDCap database without personal identifiers. Quantitative data were collected from participants, including demographic data, country of origin, native language, past medical history, and any known primary care supports. Additionally, health data, including a manually obtained blood pressure, heart rate, point-of-care capillary blood glucose, height, and weight, were recorded at a single point in time during the health screening. A single blood pressure reading was collected using manual auscultation after a minimum of a 3-minute rest period with the participant in a seated position, feet flat on the floor, and arm supported at heart level using appropriately sized cuffs. Resting heart rate was collected using a standard pulse oximeter with participants in the same position as the blood pressure reading was taken. Blood glucose was measured using a point-of-care capillary glucometer during routine health screenings. Time since last food intake was recorded as reported by participants. The glucometers used during the health screenings included the OneTouch Verio and Contour Next blood glucose monitor. Individuals with abnormal screening findings were informed of their results during the clinical screening encounter. Participants with elevated blood pressure, abnormal blood glucose values, or other clinically significant findings were referred to appropriate primary care providers. Individuals identified as high risk, including those with screening results requiring prompt medical attention, for example, hypertensive urgency with blood pressure greater than 180/120, were referred for emergency services.

### 2.3 Variables

Health variables were coded as follows. Point-of-care capillary blood glucose measurements were categorized as normal glucose range, glucose values in the prediabetic range, and glucose values in the diabetic range using American Diabetes Association fasting plasma glucose thresholds as screening thresholds [19]. Values in the prediabetic range were between 100 and 125, and glucose values in the diabetic range were greater than 126. Blood pressure measurements were categorized as normal, elevated, stage 1 hypertensive range, stage 2 hypertensive range, or hypertensive crisis range based on American Heart Association/American College of Cardiology (AHA/ACC) blood pressure thresholds [20]. These categories were used to identify participants who may require confirmatory diagnostic evaluation and do not represent confirmed diagnoses. For the purposes of the bivariate analyses, this variable was further grouped into three categories (normal, elevated, and hypertension (stage 1 or 2 range)—hypertensive crisis range). Heart rate was categorized as (<80 bpm and ≥80 bpm), where bpm stands for beats per minute. There is literature to suggest that a heart rate above >80 bpm is correlated with increased cardiovascular risk [21, 22]. BMI was categorized as underweight, normal weight, overweight, and obese, and classified using guidelines from the National Heart, Lung, and Blood Institute [23]. Risk factor variables included age (18–44, 45–64, and 65+) based on cardiovascular risk factors and gender (male, female, other, not sure, and prefer not to answer) [24, 25]. Country of origin was recoded based on the World Health Organization’s List of Regions as follows: African Region (Burundi, Central African Republic, Chad, Congo, Eritrea, Ethiopia, Kenya, Mali, Nigeria, Rwanda, South Sudan, Sudan, Tanzania, Uganda, and Zambia), Region of the Americas (Colombia, Cuba, Ecuador, El Salvador, Guatemala, Nicaragua, and Venezuela), South-East Asian Region (Bangladesh and Burma/Myanmar), European Region (Palestine, Turkey, and Ukraine), Eastern Mediterranean Region (Afghanistan, Egypt, Iraq, Jordan, Somalia, Syria, and Yemen), and Western Pacific Region (Malaysia) [26].

### 2.4 Data analysis

Data were exported from REDCap. Data were cleaned and analyzed with Statistical Package for the Social Sciences (SPSS) version 29.0. Descriptive statistics were used to characterize blood pressure measurements and point-of-care glucose values in the refugee population. The proportion of participants with blood pressure measurements in the hypertensive range and glucose values in the diabetic range was compared with estimates of hypertension and diabetes in the general U.S. population. Bivariate analysis was conducted using two-tailed Fisher’s exact, Chi-square, and independent sample *t*-tests to explore the relationship between diabetes status, blood pressure, and heart rate with the risk factors such as age, gender, region of origin, BMI, blood pressure, and diabetes status. For diabetes, we explored associations with age, gender, region of origin, BMI, and blood pressure. For blood pressure, we explored associations with age, gender, BMI, and diabetes status. For heart rate, we looked at associations with age, gender, region of origin, BMI, blood pressure, and diabetes.

For past medical history, an independent sample *t*-test explored the relationship between a medical history of diabetes (No previous diagnoses and Previously diagnosed) and blood glucose level (continuous). Fisher’s exact tests explored the association between having a history of hypertension (No previous diagnoses and Previously diagnosed) and blood pressure (Normal, Elevated, and High). An independent sample *t*-test was also used to explore the association between having a past medical history of both hypertension and diabetes (no previous diagnoses and previously diagnosed), with blood glucose level (continuous). Fisher’s exact test also explored the association between having a past medical history of both hypertension and diabetes, with blood pressure, and having a past medical history of both hypertension and diabetes with current diabetes status. In addition, Fisher’s exact explored having a past medical history of hypertension and diabetes with a current diabetes status.

## 3. Results

### 3.1 Descriptive analysis

Table 1 shows the demographic characteristics of participants. There were more females (295, 55.8%) than males (232, 43.9%), and most participants were 18–44 years old (369, 69.8%). The most common region of origin was the Eastern Mediterranean Region (208, 39.3%). Most participants arrived in the U.S. in 2024 (312, 59.0%). For primary care providers (PCPs), most did not have a PCP before arriving in the U.S. (350, 66.2%) or after arriving in the U.S. (445, 84.1%).

**Table 1:** Demographic characteristics for health screening participants (*N* = 529).

CHARACTERISTIC	*N* (%)
**Gender**	
Male	232 (43.9)
Female	295 (55.8)
Other/not sure	2 (0.4)
Missing	0 (0.0)
**Age**	
18–44 years old	369 (69.8)
45–64 years old	122 (23.1)
65+ years old	38 (7.2)
Missing	0 (0.0)
**Region of origin**	
African Region	191 (36.1)
Region of the Americas	66 (12.5)
South-East Asian Region	43 (8.1)
European Region	15 (2.8)
Eastern Mediterranean Region	208 (39.3)
Western Pacific Region	1 (0.2)
Missing	5 (0.9)
**Arrival year**	
2019	2 (0.4)
2020	0 (0.0)
2021	2 (0.4)
2022	10 (1.9)
2023	140 (26.5)
2024	312 (59.0)
2025	51 (9.6)
Missing	11 (2.1)
**PCP before the U.S.**	
Yes	122 (23.1)
No	350 (66.2)
Not sure	40 (7.6)
Not applicable	15 (2.8)
Missing	2 (0.4)
**PCP in the U.S.**	
Yes	31 (5.9)
No	445 (84.1)
Not sure	19 (3.6)
Not applicable	32 (6.0)
Missing	2 (0.4)

Supplementary File 1 shows the language distribution for participants. The most reported languages were Arabic (16.3%), Swahili (15.7%), and Somali (13.6%). See Supplementary File 1 for additional details.

Table 2 shows that most participants (61.1%) had a blood pressure reading that was above the threshold for “normal.” This includes 53.2% of participants who had a blood pressure reading falling within the hypertension stages or hypertensive crisis. Among all participants, 51.6% had a heart rate reading less than 80 bpm and 42.3% had 80 bpm or greater. The BMI of 52.4% of participants fell within the overweight or obese ranges. Based on a point-of-care capillary glucose level, 81.1% of participants fell within the normal range, and 15.7% were within the prediabetes and diabetes ranges.

**Table 2:** Clinical measurements of health screening participants (*N* = 529).

CHARACTERISTIC	*N* (%)
**Blood pressure**	
Normal range	206 (38.9)
Elevated range	42 (7.9)
Stage 1 hypertensive range	167 (31.6)
Stage 2 hypertensive range	111 (21.0)
Hypertensive crisis range	3 (0.6)
Missing	0 (0.0)
**Heart rate**	
<80 bpm	273 (51.6)
≥80 bpm	224 (42.3)
Missing	32 (6.0)
**BMI**	
Underweight	27 (5.1)
Normal weight	185 (35.0)
Overweight	158 (29.9)
Obese	119 (22.5)
Missing	40 (7.6)
**Diabetes**	
Normal range	429 (81.1)
Prediabetes range	46 (8.7)
Diabetes range	37 (7.0)
Missing	17 (3.2)

A total of 53.2% of participants were either in stage 1 or 2 hypertension range or in the hypertensive crisis range. See Figure 1 for more detail.

**Figure 1:**
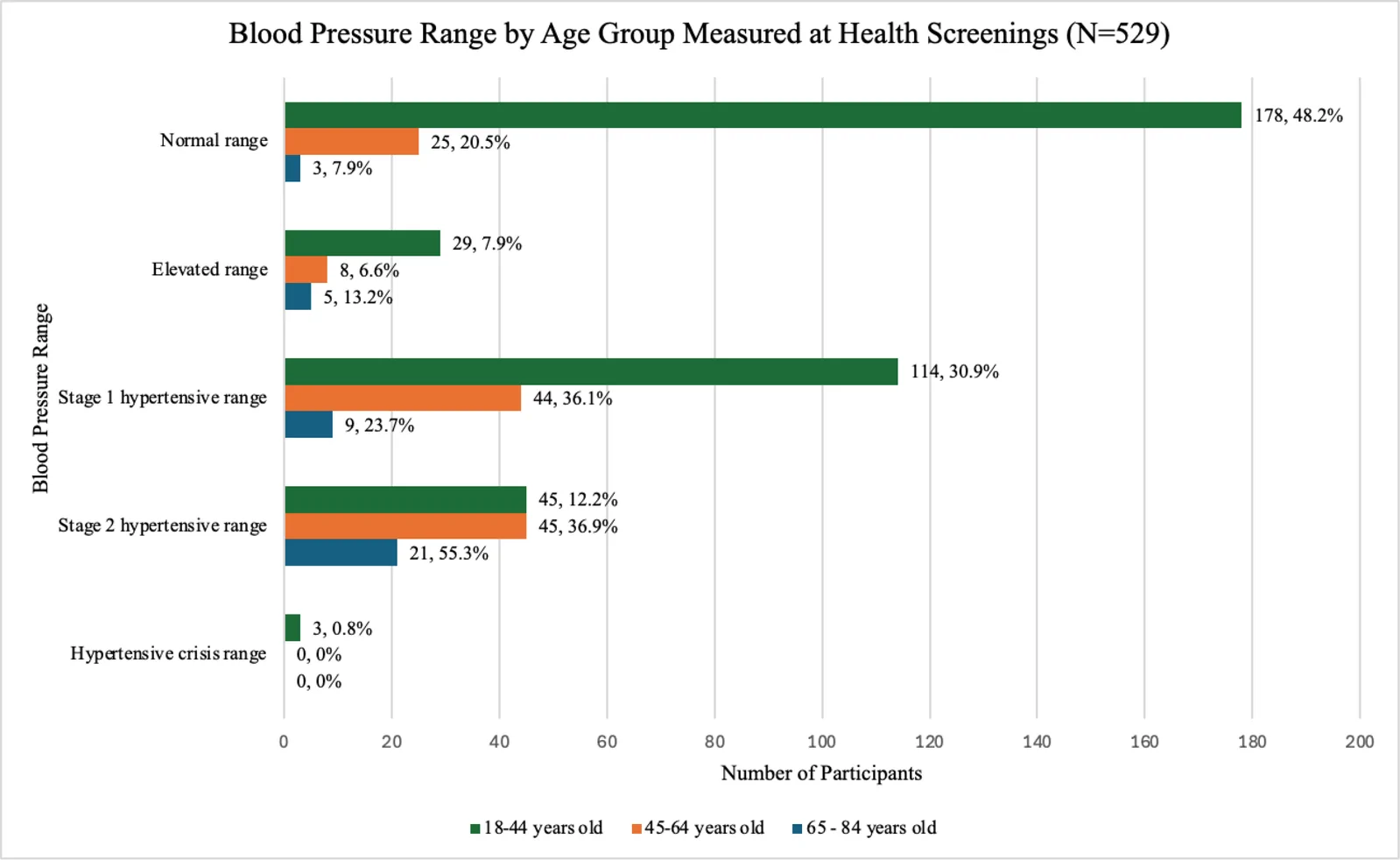
Blood pressure measurement range by age group measured at health screenings (*N* = 529).

Most participants fell into the normal range or prediabetes range. See Figure 2 for more detail.

**Figure 2:**
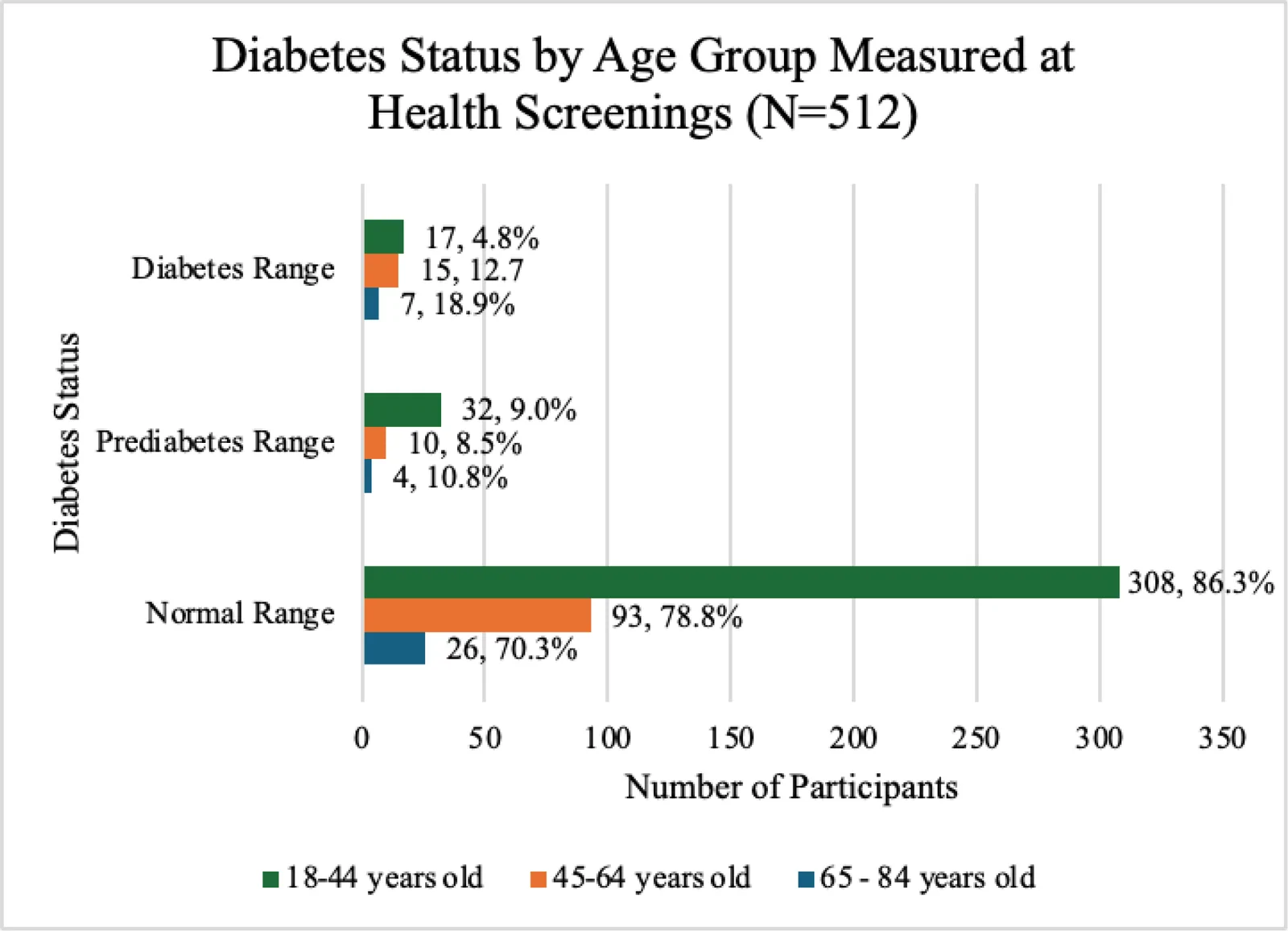
Point-of-care capillary glucose values by age group measured at health screenings (*N* = 512).

### 3.2 Bivariate analysis for health measures

#### 3.2.1 Diabetes, blood pressure, and heart rate

Fisher’s exact test results revealed an association between diabetes status and age (*p* = 0.001) as well as diabetes status and blood pressure (*p* = 0.039). No associations were found between diabetes status and gender, region of origin, or BMI. Fisher’s exact test results revealed an association between blood pressure and age (*p* < 0.001), gender (*p* < 0.001), and BMI (*p* < 0.001), Fisher’s exact test results revealed an association between heart rate and gender (*p* = 0.021), and Chi square results revealed an association between heart rate and diabetes status [*X*^2^(2) = 7.30, *p* = 0.03]. No significant associations were found between heart rate and age, region of origin, BMI, or blood pressure. See Table 3 for more details.

**Table 3:** Significant results for Fisher’s exact and Chi-square tests looking at diabetes status, blood pressure, and heart rate by risk factors (*N* = 529).

		*N* (%)		FISHER’S EXACT VALUE *CHI-SQUARE VALUE (DF)	*P*-VALUE	CRAMER’S V, *P*-VALUE *ODDS RATIO, 95% CI
	**Diabetes status**					
	Normal	Prediabetes range	Diabetes range	16.92	0.001	0.13, *p* = 0.002
**Age**						
18–44 years old	310 (72.3)	32 (69.6)	15 (40.5)			
45–64 years old	93 (21.7)	10 (21.7)	15 (40.5)			
65+ years old	26 (6.1)	4 (8.7)	7 (18.9)			
Valid cases	512					
**Blood pressure**				9.69	0.039	0.10, *p* = 0.05
Normal	166 (38.7)	23 (50.0)	11 (29.7)			
Elevated	39 (9.1)	0 (0.0)	1 (2.7)			
High blood pressure	224 (52.2)	23 (50.0)	25 (67.6)			
Valid cases	512					
	**Blood pressure**					
	Normal	Elevated	High blood pressure	53.37	<0.001	0.22, *p* < 0.001
**Age**						
18–44 years old	178 (86.4)	29 (69.0)	162 (57.7)			
45–64 years old	25 (12.1)	8 (19.0)	89 (31.7)			
65+ years old	3 (1.5)	5 (11.9)	30 (10.7)			
Valid cases	529					
**Gender**				16.22	<0.001	0.18, *p* < 0.001
Male	69 (33.5)	18 (43.9)	145 (51.8)			
Female	137 (66.5)	23 (56.1)	135 (48.2)			
Valid cases	527					
**BMI**				29.00	<0.001	0.17, *p* < 0.001
Underweight	14 (7.3)	4 (10.0)	9 (3.5)			
Normal weight	93 (48.2)	15 (37.5)	77 (30.1)			
Overweight	58 (30.1)	11 (27.5)	89 (34.8)			
Obese	28 (14.5)	10 (25.5)	81 (31.6)			
Valid cases	489					
	**Heart rate**					
	<80 bpm	≥80 bpm		*5.49	0.012	*1.54, 1.07–2.21
**Gender**						
Male	131 (48.2)	84 (37.7)				
Female	141 (51.8)	139 (62.3)				
Valid cases	495					
**Diabetes status**				*7.30 (2)	0.026	0.12, *p* = 0.03
Normal	231 (86.5)	187 (84.6)				
Prediabetes	24 (9.0)	12 (5.4)				
Diabetes	12 (4.5)	22 (10.0)				
Valid cases	488					

#### 3.2.2 Past medical history of hypertension and diabetes

When asked about past medical history, most respondents reported that they were not sure or that they had not been diagnosed with any diseases (234, 44.2%). A past diagnosis of hypertension was most reported (90, 17.0%), followed by a past diagnosis of diabetes (37, 7.0%). A total of 22 (4.2%) participants reported a past diagnosis of both hypertension and diabetes. Other chronic conditions were most common (134, 25.3%) (see Figure 3).

**Figure 3:**
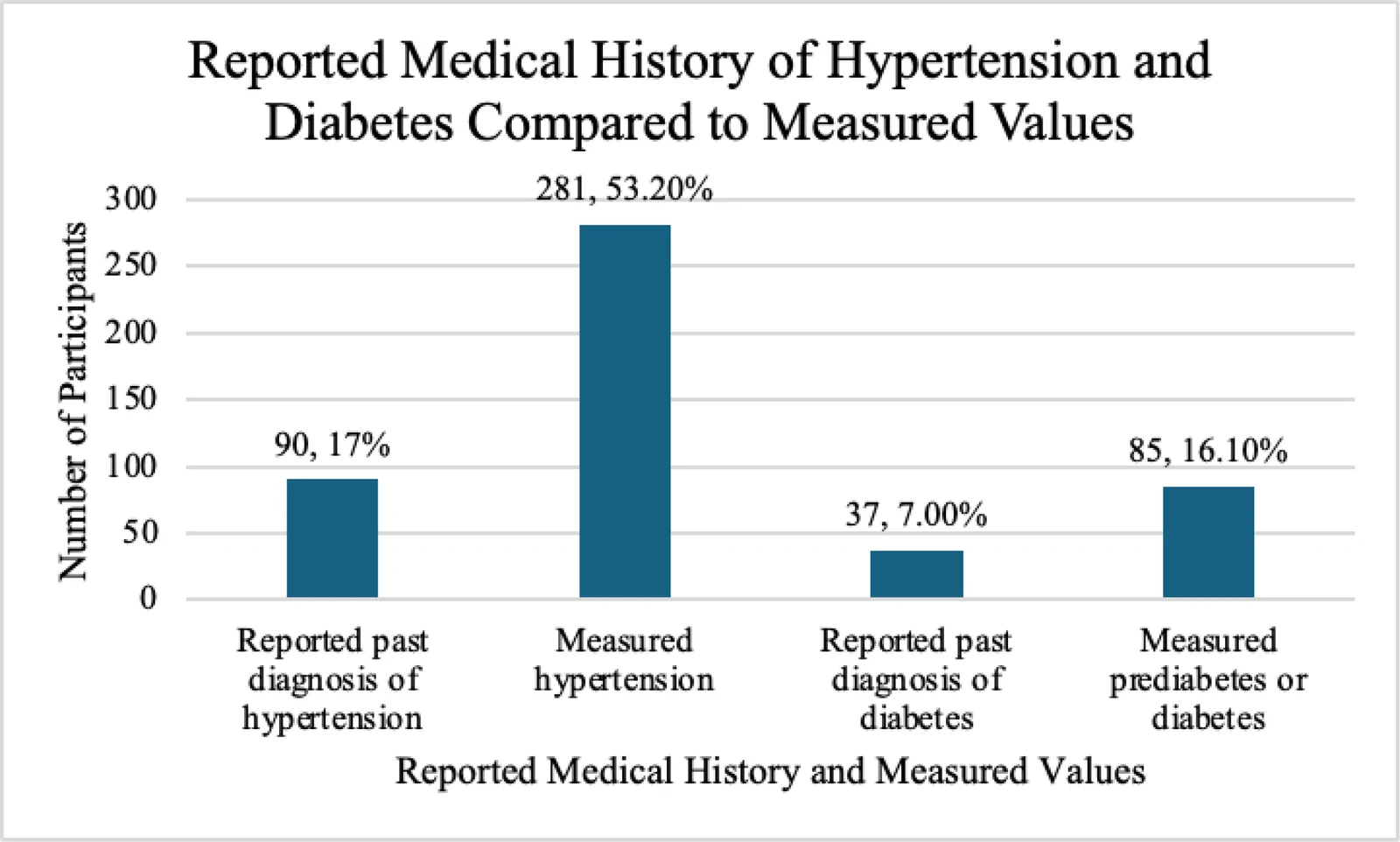
Reported medical history of hypertension and diabetes compared to values measured at health screenings.

However, the results of the health screening measurements suggested a higher burden of cardiometabolic abnormalities. Although only 17.0% of participants reported a prior diagnosis of hypertension, 61.1% had blood pressure readings above the normal threshold (Table 2), including 53.2% in the hypertension stages or hypertensive crisis. Similarly, while 7.0% reported a prior diagnosis of diabetes, 16.1% had point-of-care capillary glucose values in the prediabetes or diabetes ranges (Table 2).

Previous diagnosis of diabetes and blood glucose level were found to be associated (*t*(514) = −10.123, *p* < 0.001, mean difference –70.99, 95% CI –84.77, −57.21). Having a past medical history of hypertension was associated with blood pressure (Fisher’s exact test = 42.065, *p* < 0.001). A history of diabetes and hypertension together was associated with both blood glucose (*t*(514) = −4.812, *p* < 0.001, mean difference −45.58, 95% CI –64.19, −26.97) and blood pressure (Fisher’s exact = 13.927, *p* < 0.001, Cramer’s V = 0.149). Having a past medical history of diabetes and hypertension together was also associated with diabetes status (Fisher’s exact = 17.299, *p* < 0.001, Cramer’s V = 0.24). Fisher’s exact test results also revealed that a medical history of diabetes was associated with current diabetes status (Fisher’s exact = 43.663, *p* < 0.001, Cramer’s V = 0.390).

Table 4 shows results for two Fisher’s exact tests, illustrating the number of people in our population who were not previously diagnosed with either hypertension or diabetes, and then diagnosed through the health screenings.

**Table 4:** Fisher’s exact test results illustrating test results at the health screenings (*N* = 529).

		*N* (%)		FISHER’S EXACT VALUE	*P*-VALUE	CRAMER’S V, *P*-VALUE
	**Blood pressure**			42.07	<0.001	0.27, *p* < 0.001
	Normal	Elevated	High			
**Medical history of hypertension**						
No previous diagnosis	194 (44.2)	39 (8.9)	206 (46.9)			
Previous diagnosis	12 (13.3)	3 (3.3)	75 (83.3)			
Valid cases	529					
	**Diabetes status**			45.69	<0.001	0.40, *p* < 0.001
	Normal	Prediabetes range	Diabetes range			
**Medical history of diabetes**						
No previous diagnosis	411 (86.2)	45 (9.4)	21 (4.4)			
Previous diagnosis	18 (51.4)	1 (2.9)	16 (45.7)			
Valid cases	512					

## 4. Discussion

The findings of this paper provide insights into the non-communicable disease burden and risk factors for hypertension and diabetes among refugees in Syracuse, NY. Our evaluation makes a significant contribution to existing literature by assessing the effects of risk factors on measures of blood pressure, point-of-care capillary glucose, and, importantly, heart rate.

### 4.1 Current burden of disease

Through the health screenings conducted between May 2023 and March 2025, we were able to identify participant demographics, BMI distribution, point-of-care capillary glucose levels, vital signs, and past medical history. The demographics reveal the diverse backgrounds of the refugee population in Syracuse, NY. In terms of BMI, 52.4% of participants were either overweight or obese, compared to 73.1% of U.S. adults [27]. Additionally, the prevalence of obesity in our participants was 22.5%, compared to 42.4% of US adults [27]. There is literature to suggest that increased length of stay in the U.S. is associated with increased rates of obesity and elevated BMI, thereby increasing cardiovascular risk factors [28]. Factors that may influence this association include acculturation in diet with increased intake of processed and calorie-dense foods, food availability, environment, and cultural influences [29]. These findings demonstrate that non-communicable chronic disease, including obesity, affects refugee populations and emphasize the importance of early nutrition and physical activity education during the resettlement process and continued monitoring post-resettlement.

For hypertension in this population, notably, the findings reveal that 61.1% of participants had a blood pressure reading above the threshold for “normal.” This figure is consistent with a study among refugees in San Antonio, Texas [30] and is higher than the U.S. national population prevalence of hypertension at 47.7% [31]. Factors that could be influencing these elevated blood pressure readings include physical and emotional stress during the early resettlement process, white coat hypertension, and the use of only one blood pressure reading. Even considering these limitations, the high proportion of participants with a screening blood pressure measurement above the normal range highlights the potential burden of cardiometabolic risk in this population and the importance of confirmatory evaluation. This underscores the need for enhanced disease surveillance.

The discrepancy between self-reported past medical history and screening measurements suggests a substantial burden of previously unrecognized cardiometabolic risk within this refugee population, highlighting the value of early health screenings, health literacy education, and preventative health engagement during the resettlement process.

Glucose levels were measured, and although promising that 81.1% of participants fell within the normal range, a notable number of participants fell within the prediabetes (8.7%) and diabetes (7.0%) range. A single elevated blood sugar measurement does not yield the diagnosis of diabetes, but it can serve as an important early indicator of an underlying diagnosis. A slight majority of participants had heart rate <80 bpm (51.6%), and 42.3% of participants had a heart rate ≥80 bpm. The heart rate cutoffs were established based on previous literature that supported elevated heart rate as a risk factor for arterial hypertension [10]. Most studies on hypertension have reported that a heart rate greater than 80–85 bpm offers an increased cardiovascular and mortality risk [10].

### 4.2 Clinical associations

#### Diabetes

The association between diabetes and hypertension has been well documented in literature, with these conditions being closely linked by pathophysiology and clinical outcomes, with studies reporting prevalence of hypertension in adults with diabetes between 65 and 75% [32]. A Fisher’s exact analysis of our data also emphasizes a significant association between blood glucose readings in the diabetes range and both blood pressure (Fisher’s exact = 9.6, *p*-value = 0.039) and age (Fisher’s exact = 16.92, *p*-value = 0.0014), in line with existing literature. Our findings support previous research on the important contribution of poor glycemic control and elevated blood pressure [33, 34].

#### Blood Pressure

Within our cohort, blood pressure was found to have significant associations with several demographic and clinical measurements. A Fisher’s exact analysis between blood pressure and age of participants showed a statistically significant association (Fisher’s exact = 53.37, *p*-value < 0.001). Associations were also found between blood pressure and gender (Fisher’s exact = 16.22, *p*-value < 0.001), as well as blood pressure and BMI (Fisher’s exact = 29, *p*-value < 0.001). Literature suggests that men in the general population are more likely to have hypertension than females, due to both biological and behavioral factors, although limited studies have looked at refugee populations specifically [28, 35]. Our results support the findings in previous research, highlighting the association between blood pressure and age, BMI, and gender in refugee populations [36, 37].

#### Heart Rate

Heart rate was found to be associated with gender and blood glucose levels in the diabetes ranges within our cohort. Furthermore, within our cohort, of the participants who had a heart rate ≥80 bpm, 62.3% were female and 37.7% were male. Previous literature suggests a relationship between heart rate and gender, noting that females on average have a 7% higher heart rate than males [38, 39]. Additionally, Fisher’s exact and Chi-square tests showed an association between heart rate and diabetes. Within our cohort, the data suggests that heart rate was associated with elevated glucose measurements, especially in the diabetic range.

Although there have been some studies documenting this in literature, the association between heart rate and both diabetes and undiagnosed diabetes should be further explored [40]. This link could suggest that heart rate be an early, non-invasive indicator of glycemic abnormalities, especially in the appropriate clinical context, but additional research is needed to further document this link.

### 4.3 Comparison with U.S. National estimates

It is useful to contextualize the blood pressure and point-of-care capillary glucose in relation to that of U.S. National estimates. In terms of elevated blood pressure readings, our analysis found that 52.6% of participants had a single screening blood pressure measurement within the Stage 1 or Stage 2 hypertensive range based on AHA/ACC blood pressure thresholds. In the U.S., approximately 48.1% or 119.9 million adults have a blood pressure at or above 130/80 or are currently taking blood pressure lowering medications [41]. Our statistic includes only individuals in our cohort in the hypertensive range on a single blood pressure measurement and does not consider any individuals who may be on blood pressure lowering medications. Therefore, the true presence of blood pressures in the hypertensive range may be higher than we report; however, our measurement is based on a single reading.

Most of our participants were in the age range between 18 and 44 years. 43.9% participants within this age group had single blood pressure readings in the hypertensive range. This is in comparison to 29.8% of people in the general U.S population [42]. For participants between the ages of 45 and 64, our cohort had 72.95% with blood pressure readings in the hypertensive range compared to 64.4% in the general U.S. cohort. For adults in our cohort 65 years and above, 78.9% of participants had blood pressure readings in the hypertensive range. This is in comparison to approximately 81.0% of the U.S. population between the ages of 65 and 74 years. While our blood pressure measurements are not aimed at diagnosing our participants with hypertension based on a single reading, these results emphasize the prevalence of elevated blood pressure in our refugee cohort, nonetheless.

Based on our point-of-care glucose capillary levels, 9.0% of our participants had blood glucose levels between 100 and 125. Furthermore, 7.2% of our participants had blood glucose levels greater than 125, with 3.6% with blood glucose level >200. The prevalence of diabetes in adults in the U.S. is estimated to be approximately 12.0% [43]. While our point-of-care capillary glucose levels are unable to unequivocally establish a diagnosis of diabetes, it highlights that hyperglycemia is present in our refugee cohort, and further screening and diagnostic testing may be warranted. There are studies that have examined the utility of random glucose values as a case-finding strategy to detect undiagnosed diabetes, specifically random glucose ≥100 mg/dL as a highly sensitive and specific test to identify individuals in need of diabetes screening [44]. Thus, 16.1% of our participants with random glucose ≥100 mg/dL may benefit from further diagnostic evaluation.

### 4.4 Population health impacts

Diabetes mellitus and hypertension represent clinically significant and interrelated chronic conditions that disproportionately affect refugee populations. Refugees often arrive with a high burden of undiagnosed or poorly controlled cardiometabolic diseases due to prolonged disruptions in healthcare access, food insecurity, chronic psychosocial stressors, and limited, if any, opportunities for preventive care prior to resettlement [45, 46]. Additional barriers, including language, limited health literacy, and cultural differences in treatment of health conditions, further disrupt timely diagnosis and long-term management of both acute and chronic conditions following resettlement. Current U.S. refugee health guidance recommends a comprehensive Refugee Health Assessment (RHA) after arrival to identify and manage chronic conditions [8]. This early health screening program evaluated here provides an additional opportunity to identify elevated blood pressure, elevated blood glucose values, and elevated resting heart rate as important cardiometabolic risk factors. Elevated resting heart rate and tachycardia represent clinically important physiologic biomarkers that are hypothesized to indicate underlying autonomic dysfunction, chronic stress, or cardiovascular disease. Furthermore, elevated BMI has shown direct associations with cardiovascular disease in refugee populations [1]. Given the association between diabetes and hypertension and heart rate and diabetes, these biomarkers identify a particularly high risk and vulnerable group of individuals that underscore the importance of early identification and culturally sensitive management strategies to improve outcomes and reduce health disparities among refugee populations. These findings suggest that early community-based screening may complement existing domestic refugee health practices by facilitating earlier referrals and linkage to care for refugees with abnormal cardiometabolic screening findings. Notably, 84.1% of participants did not have an established primary care provider in the United States at the time of screening; this finding likely reflects the early transition period of resettlement, when many individuals had not yet established ongoing primary care. Participants without established primary care providers were connected to primary care as part of the resettlement healthcare process, with linkage facilitated through existing resettlement services and, following implementation of the Refugee and Immigrant Health Hub (RIHH), through RIHH staff [47]. This process provided an important opportunity to establish continuity of care soon after arrival.

As CCP health screenings routinely identified cardiometabolic risk factors in a limited health literacy population, there was demonstrable need to have a more centralized public health support for population screening and access to primary care for a vulnerable population. In 2024, the Onondaga County Health Department established RIHH, a public health nursing driven triage to support the review and health screening of all newly arrived refugees coming to Syracuse. RIHH serves as an access point into four primary care practices that are accepting refugees, with a medical triage that prioritizes the health needs of a vulnerable cohort [47].

### 4.5 Limitations

Despite its strengths, this study has several limitations. First, selection bias is possible because participant identification varied over the project period. The participants were primarily refugees resettled by CCOC Refugee Resettlement Services, Interfaith Works of Central New York, and Refugee and Immigrant Self Empowerment. If abnormalities are detected on pre-departure screening and if participants have known past medical history, then case managers and medical staff were more likely to encourage those participants to take part in the health screenings.

Second, while interpretation and translation services were employed where necessary, the nuances of patient histories may not have been fully conveyed across languages, potentially leading to variability in collected data. Limited health literacy and poor knowledge of chronic health conditions may have led to the underreporting of established diagnoses of hypertension and diabetes. Another limiting factor in this paper includes measurements at a single time point. Since health screenings are conducted typically as a one-off event instance, it is not possible to determine causality among risk factors and elevated glucose levels, blood pressure measurements in the hypertensive range, and elevated heart rate. Results may not be generalizable to the larger refugee community outside of central New York because of the aforementioned limitations. Due to the number of bivariate tests conducted, there is a possibility of Type 1 errors in our results, and since our results are exploratory, we did not apply formal multiple-testing adjustments and bivariate results should be interpreted as preliminary.

Ultimately, because this initiative and data collection were conducted independent of research intent, the findings of our evaluation are primarily intended to better characterize the health status of refugee communities, highlight the importance of early screening and detection, and inform health promotion efforts for refugees.

## 5. Conclusions

This paper identified a substantial proportion of newly arrived refugees in Syracuse, NY, with blood pressure measurements in the hypertensive range during initial health screening, highlighting the need for timely confirmatory evaluation and linkage to primary care. Although the prevalence of screening glucose values in the diabetic range was lower than that of the general U.S. population, 4.4% of participants without a previous diagnosis had glucose values consistent with the diabetes range and an additional 8.7% had values consistent with the prediabetes range, demonstrating important opportunities for early detection in a relatively young population. Elevated glucose values were significantly associated with increasing age and higher blood pressure, while blood pressure measurements in the hypertensive range were associated with increasing age, male gender, and higher BMI. Elevated resting heart rate (≥80 bpm) was also associated with female gender and glucose values in the diabetic range, suggesting its potential utility as an early marker of cardiometabolic risk. These findings underscore the value of early cardiometabolic screening to identify refugees who may benefit from confirmatory diagnostic testing, timely referral, and ongoing clinical management. Importantly, these health screenings demonstrated the need for greater public health support for population screening and access to primary care for refugees. This need directly supported the establishment of the RIHH, which has since triaged and connected hundreds of refugees to services for their respective health needs. Looking ahead, this initiative aims to continue informing best practices for refugee health promotion through health literacy education, primary care services, and sustained community partnerships.

## Data Availability

The de-identified dataset analyzed during the current project is not publicly available because it was collected as part of an institutional public health initiative and is subject to institutional data governance and ethical considerations related to projects involving refugee populations. De-identified data may be made available from the corresponding author upon reasonable request, subject to approval by the appropriate institutional authorities and applicable ethical requirements.

## Additional File

The additional file for this article can be found as follows:

Supplementary material. Language for the study population (N = 529).
