# Supplementary material for: Chronic Non-Communicable Disease Burden among Recently Arrived Refugees in Syracuse, New York
Source: Ann Glob Health. 2026 Oct 15;92(1):101. doi: 10.5334/aogh.5247 (PMC13683940; doi:10.5334/aogh.5247)
Supplement: Supplementary file 1 [file agh-92-1-5247-s1.pdf]

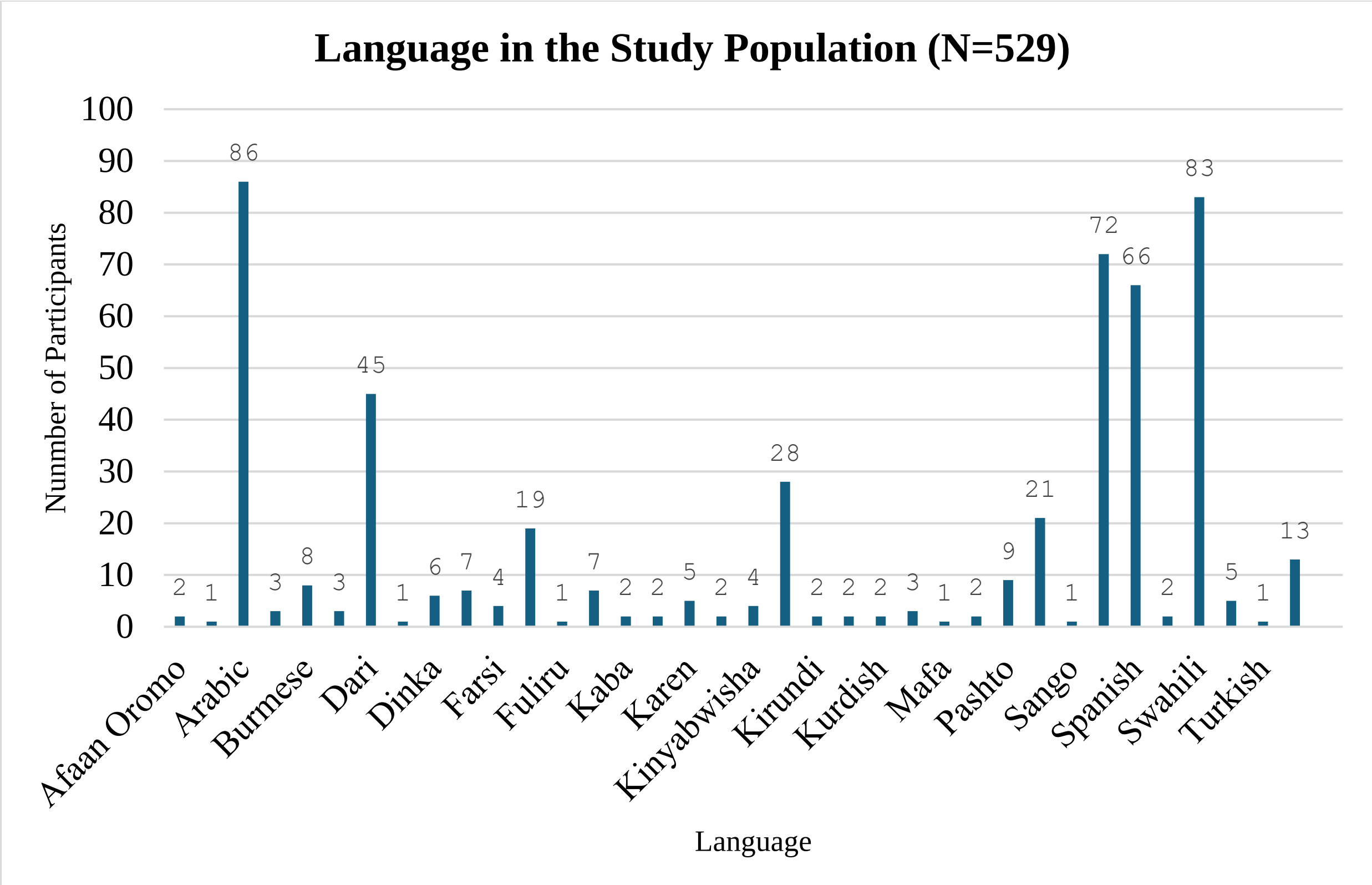

Supplementary Material: Language for the study population (N=529).
